# Improving dementia care: insights from audit and feedback in interdisciplinary primary care sites

**DOI:** 10.1186/s12913-022-07672-5

**Published:** 2022-03-17

**Authors:** Geneviève Arsenault-Lapierre, Mélanie Le Berre, Laura Rojas-Rozo, Carrie McAiney, Jennifer Ingram, Linda Lee, Isabelle Vedel

**Affiliations:** 1 Department of Family Medicine, McGill University, Montreal, Quebec Canada; 2 Lady Davis Institute for Medical Research, Jewish General Hospital, Quebec, Montreal Canada; 3 School of Public Health and Health Systems, University of Waterloo and Schlegel-UW Research Institute for Aging, Waterloo, Ontario Canada; 4 Kawartha Centre - Redefining Healthy Aging, and Senior Care Network, Central East Ontario, Peterborough, Ontario Canada; 5 Schlegel-UW Research Institute for Aging, Waterloo, ON Canada; 6 Department of Family Medicine, McMaster University, Hamilton, Ontario Canada

**Keywords:** Primary Care, Dementia, Practice Guideline, Medical Audit, Quality Improvement, Interdisciplinary Health Team

## Abstract

**Background:**

Many primary care sites have implemented models to improve detection, diagnosis, and management of dementia, as per Canadian guidelines. The aim of this study is to describe the responses of clinicians, managers, and staff of sites that have implemented these models when presented with audit results, their insights on the factors that explain their results, their proposed solutions for improvement and how these align to one another.

**Methods:**

One audit and feedback cycle was carried out in eight purposefully sampled sites in Ontario, Canada, that had previously implemented dementia care models. Audit consisted of a) chart review to assess quality of dementia care indicators, b) questionnaire to assess the physicians’ knowledge, attitudes and practice toward dementia care, and c) semi-structured interviews to understand barriers and facilitators to implementing these models. Feedback was given to clinicians, managers, and staff in the form of graphic and oral presentations, followed by eight focus groups (one per site). Discussions revolved around: what audit results elicited more discussion from the participants, 2) their insights on the factors that explain their audit results, and 3) solutions they propose to improve dementia care. Deductive content and inductive thematic analyses, grounded in causal pathways models’ theory was performed.

**Findings:**

The audit and feedback process allowed the 63 participants to discuss many audit results and share their insights on a) organizational factors (lack of human resources, the importance of organized links with community services, clear roles and support from external memory clinics) and b) clinician factors (perceived competency practice and attitudes on dementia care), that could explain their audit results. Participants also provided solutions to improve dementia care in primary care (financial incentives, having clear pathways, adding tools to improve chart documentation, establish training on dementia care, and the possibility of benchmarking with other institutions). Proposed solutions were well aligned with their insights and further nuanced according to contextual details.

**Conclusions:**

This study provides valuable information on solutions proposed by primary care clinicians, managers, and staff to improve dementia care in primary care. The solutions are grounded in clinical experience and will inform ongoing and future dementia strategies.

**Supplementary Information:**

The online version contains supplementary material available at 10.1186/s12913-022-07672-5.

## Background

Provincial and national dementia guidelines have been published in Canada for improving the care of persons with dementia and their caregivers [1–4]. These guidelines identify interdisciplinary primary care sites, with the support from specialized services when needed, as the cornerstone in dementia care [5]. Furthermore, many primary care clinicians are willing and ready to assume a key role in dementia care [6, 7]. However, some clinicians face difficulties in providing dementia care in primary care settings [8]. Various studies have explored barriers in the management of dementia in primary care that included failure to recognize symptoms, lacking information and understanding of the disease, lacking support to the primary care clinicians, diagnosis uncertainty, dementia complexity, and lack of time [8–10].

Practice guidelines can be difficult to translate into clinical practice [11]. Clinicians, including Canadian clinicians, may perceive these guidelines as both enablers and barriers to the identification, assessment, and management of dementia [8]. There is a need to better support the implementation of dementia guidelines in primary care sites in Canada [1].

Many different implementation strategies exist to support dissemination and scale up of innovative models of care [12]. One of these strategies, the audit and feedback (A&F) approach, can be useful to improve clinical practice [12, 13], especially when repeated over several cycles [14]. Furthermore, clinicians commonly see A&F as a positive experience [13]. Canadian family physicians consider it an essential part of medical professionalism, important for maintaining their accountability [15]. Because A&F aims at improving clinicians’ adherence to guidelines [16, 17], through action plans [18] and recommendations for dissemination to future primary care sites and provincial policies, this strategy represents a good option.

## Methods

### Aim

The overarching aim of our study was to describe the responses of clinicians, managers and staff of primary care sites that have implemented various models of dementia care when presented with audit results, their insights on the factors that explain the audit results, their proposed solutions to improve dementia care and how these insights and solutions align to one another. More specifically, we had the following three sub-objectives: 1) describe what audit results elicited more discussion from the participants, 2) describe their insights on the factors that explain their results, and 3) describe solutions proposed they would like to implement to improve dementia care in primary care.

### Design

We conducted a one-cycle A&F study [18, 19] in eight interdisciplinary primary care sites in Ontario, Canada, that had previously implemented models of dementia care. We used the SQUIRE 2.0 reporting guidelines for quality improvement studies [20] to guide the writing of this manuscript.

### Setting and participating primary care sites

This study is part of a multi-provincial Canadian study taking place in Quebec, Ontario and New Brunswick funded through the Canadian Consortium on Neurodegeneration in Aging (CCNA) [21]. This larger study aims to examine innovative primary care models for persons with dementia. For the current study, we focused on eight purposefully sampled primary care sites (hereafter referred to as ‘sites’) in Ontario.

As per the larger study protocol, the sites were selected because they were interdisciplinary primary care sites, they had previously implemented an innovative primary dementia care model, they were knowledgeable about existing dementia guidelines, and they were motivated to improve dementia primary care for their population. Four of the eight sites had previously implemented an embedded-assessor model, two sites had implemented a collaborative memory clinic model, and two sites had implemented a combination of both in a hybrid model (see Additional file 1 for details on implemented models as well as a timeline of the implementation of the models, the audit, the feedback, and focus group discussions).

### Audit

The audit was performed after the models of dementia care had been implemented in the eight primary care sites. To conduct a comprehensive audit at the eight clinics, we used three sources of data.

First, we carried out a retrospective chart review of 35 patients per site with a diagnosis of dementia who had a visit during a nine-month period (October 1, 2015 to July 1, 2016) to obtain information on adherence to Canadian recommendations of dementia care [2, 4]. These recommendations translated into 10 indicators, composing a validated quality of follow-up score [21] (specifically, documented assessment of cognition, functional status, behavioural and psychological symptoms of dementia, weight, driving status, caregiver’s needs, home care needs, community service needs, absence of anticholinergic medication and management of dementia medications). In addition, indicators of continuity of primary care and medication management (specifically, proportion of patient with a new diagnosis during the 9-month period; proportion of patients with a new diagnosis from a physician from the site, from a specialist or during a hospitalization; average number of visits during the period per patient; average number of notes recorded in each chart; proportion of patients with a referral to an external memory clinic during the period and justification of referrals; proportion of patients with dementia treated with dementia medications; proportion of new dementia medications initiated by family physician at the site; proportion of new dementia medications initiated by specialists; and proportion of patients treated with antipsychotics during the study period) were measured along with patients’ characteristics (specifically, mean age, proportion of women, and proportion of patients living alone).

Second, using a validated questionnaire [22], distributed in 2017, we measured the knowledge, attitudes, and practice (KAP) of 100 family physicians and nurse practitioners with regards to dementia care and initiatives. The details of the questionnaire construction, validation and distribution are presented elsewhere [21]. The questionnaires measured five domains as they relate to their KAP: perceived competency and knowledge related to dementia, attitudes toward dementia, practices in terms of cognitive evaluation; attitude toward their collaboration with other healthcare professionals in the site and attitudes toward the dementia care models.

Finally, in 2017, we conducted interviews with 30 clinicians and managers to identify facilitators and barriers associated with the implementation of the dementia care models described above. The details for this method are reported elsewhere [21].

### Feedback and focus groups

For the feedback, we compiled descriptive results from the audit (number, percentage, mean, standard deviation, and range), with a focus on chart review results, into site-specific PowerPoint presentations. The site-specific results for the chart review were compared to the average for the eight participating sites and presented in bar graphs and tables. Results from the KAP questionnaire (in bar graphs) and the interviews (in point form) were provided globally, not site specifically, to avoid putting the participants’ confidentiality at risk (see Additional file 2 for a template of feedback presentations).

In June 2019, the healthcare clinicians, managers, and administrative staff of each site were invited to a feedback presentation (one presentation per site). To increase the effectiveness of the feedback provided, discussions were facilitated by respected experts [13, 14]: a public health physician and researcher (IV) and a geriatrician (JI), or a researcher in dementia care (CM), using an oral presentation format with visual support (see Additional file 2) and detailing the formal targets included in the audit.

Immediately following each feedback presentation, we conducted a focus group. The discussion guide, specifically developed for this study (Additional file 3), included the following questions: *which results did you find useful / interesting / expected / surprising?*; *what are the key elements that explain these results?*; *what changes in terms of results would you like to see when this evaluation is performed in the future?*; and *what would help your site to achieve these changes/goals?* Focus group discussions were recorded and transcribed verbatim.

### Analysis

For the first sub-objective (to describe what audit results elicited more discussion from the participants), we performed a content analysis [23] of the focus group discussions. One author (MLB) first read the transcriptions and created result-specific codes based on a pre-established list of codes from the three sources of data collected during the audit (indicators from the chart review, scores from the questionnaire, and findings of the interviews). These codes were then discussed and confirmed with two other authors (GAL, LRR). A final list of codes was established and a count by codes (at least one mention) was reported for each site and overall. A count of codes was performed by one author (MLB) and verified by another (GAL).

For the second and third objectives (to describe the participants’ insights on the factors that explain the audit results and the solutions proposed by the participants to improve dementia care), we performed an inductive thematic analysis [24] with Qualitative Data Analysis®, lite version. One author (MLB) coded line-by-line all transcripts. Each code was then discussed and revised by three other authors (IV, GAL, LRR). Codes were organized in thematic networks [25] and discussed among all authors.

We used and adapted the causal pathway models theory [26] to guide our analyses of the overarching aim (to describe the responses of clinicians, managers and staff of primary care sites that have implemented various models of dementia care when presented with audit results and how their insights align with the solutions). The reasoning for using this theory was to increase the likelihood that the solutions proposed by the participants achieve the intended change, by exploring how audit results discussed, factors that explain them, and solutions proposed are linked to one another [26]. By doing this, we aim to inform the improvement of implemented models of dementia care [26]. More specifically, we followed loosely the method proposed by Lewis [26] for grounding implementation research in causal pathway models: a) identify outcomes (proximal: audit results; distal: improvement of dementia care in primary care), b) identify factors that explain these results, c) generate solutions, and d) articulate modifiers (or influences on the factors/specifications on the solutions). The audit results and overall outcome of dementia care improvement are based in Canadian clinical guidelines (as described in [21]). The other steps were entirely based on the discussions during the focus groups with the participants. Each audit result was first linked with a factor, then each factor was linked with a solution and influences on the factors and specifications on the solutions were highlighted.

To ensure trustworthiness (credibility, transferability, dependability, and confirmability) of our study [27], we followed several strategies, including analyzing the data by three research team members and discussing this analysis with all research members, providing a clear description of our population, methods, and findings, as well as keeping records of our data analysis process and decisions made.

### Findings

#### Description of the participants taking part in the focus groups

The participants’ characteristics varied between sites, relative to the site’s size and team composition. There were 63 participants in the focus groups: 34 family physicians, five nurse practitioners and nine administrative or managerial staff. On average, eight participants per site took part in the focus groups (see Table 1).

**Table 1:** Description of focus groups participants by primary care sites

Site	101^1^	102^1^	103^1^	104^1^	105^3^	106^2^	107^4^	108^2^	Total
Family physicians	15	4	2	3	1	4	3	2	**34**
Nurse practitioners	1	1	0	1	1	1	0	0	**5**
Nurses	1	1	0	1	1	1	1	1	**7**
Other healthcare professionals	0	0	0	0	2	2	0	4	**8**
Managers	1	0	0	0	2	0	0	1	**4**
Administrative staff	0	0	0	1	0	3	0	1	**5**
Total	**18**	**6**	**2**	**6**	**7**	**11**	**4**	**9**	**63**

Legend: This table represents a count of participants to the focus groups in each site by type of healthcare professionals. 1 denotes that the site had implemented an embedded-assessor model, 2 denotes that the site had implemented a collaborative memory clinic, 3 denotes that the site had implemented a hybrid model of dementia care (combining embedded-assessor and a collaborative memory clinic); 4 denotes that the site had an embedded-assessor model at the time of the audit but had integrated a collaborative memory clinic model at the time of the feedback

#### First sub-objective: Describe what audit results elicited more discussion from the participants

Because the participants did not discuss each audit result specifically, but rather took a global approach to discussing their audit results, we were not able to count the number of times a specific result was discussed but rather we counted the number of times the codes as they relate to audit results were discussed. For example, participants discussed caregiver’s needs as the documentation of their assessment, the importance of the assessment itself or even the barriers and facilitators to performing a caregiver’s needs evaluation, therefore, we created the code “Caregiver’s needs”. Similarly, the participants discussed the rates of referrals to memory clinics, their appropriateness, or the barriers and facilitators associated with referrals to external memory clinics, therefore, we created the code “Referrals to external memory clinics”, and so on. As such, we created 16 codes as they relate to the audit results (listed in Table 2).

**Table 2:** Count of codes as they relate to audit results discussed during the focus groups by primary care sites

	101	102	103	104	105	106	107	108	N of sites
Referrals to external memory clinics	√	√	√	√	√	√	√	√	**8**
Cognitive testing	√	√	√	√	√	√	√	√	**8**
Caregiver’s needs	X	√	√	√	√	√	√	√	**7**
Diagnosis	X	X	√	√	√	√	√	√	**6**
Number of contacts / visits	X	√	√	√	√	X	√	√	**6**
Quality of follow-up	√	√	X	X	√	√	√	√	**6**
Alzheimer’s society	X	√	√	√	√	√	√	X	**6**
Dementia medications	√	X	√	√	√	X	√	√	**6**
Functional status	√	X	√	X	√	X	√	√	**5**
Driving assessment	X	√	√	X	√	X	√	√	**5**
Weight assessment	X	√	√	X	√	X	X	√	**4**
KAP scores	X	√	X	X	X	√	X	√	**3**
Prescription of antipsychotics	X	X	√	√	X	X	√	X	**3**
Prescription of anticholinergics	X	X	X	X	X	X	√	√	**2**
Home care services	X	X	X	X	X	X	√	X	**1**
Evaluation of BPSD	X	X	X	X	X	X	X	X	**0**
**N of results**	**5**	**9**	**11**	**8**	**11**	**7**	**13**	**12**	

Legend: This table represents the count of codes as they relate to the audit results discussed by the participants in the focus groups. The codes as they relate to the audit results listed in the first column were created using a content analysis. The tick marks mean that a particular code as it relates to the audit result was discussed at least once by the participants during the focus group. The X marks mean that the code as it relates to the audit result was not discussed. N of results is a count of codes as they relate to the audit results discussed by site; N of sites is a count of sites that discussed the audit results. BPSD: Behavioral and Psychological Symptoms in Dementia, KAP: Knowledge attitudes and practice

Sites discussed an average of 9.5 audits results. Some sites discussed up to 13 different audit results, whereas one site discussed as few as five audit results.

All sites discussed referrals to external memory clinic and cognitive testing. All but one sites discussed audit results as they relate to caregivers’ needs. Six sites discussed diagnosis, number of contacts or visits, and dementia medications and more than half the sites discussed quality of follow-up, in general or its specific indicators: Alzheimer societies, functional status, driving and weight assessments, making these audit results most discussed by the sites.

Only two to three sites discussed KAP scores, prescription of antipsychotics, and anticholinergic medications. Finally, only one site discussed home care services and no site discussed behavioral and psychological symptoms of dementia (BPSD) evaluation.

#### Second sub-objective: Describe the participants’ insights on the factors that explain audit results

Participants shared many insights to explain their audit results. These insights were grouped into organizational factors and clinicians’ factors.

#### Organizational factors that explain the primary care sites’ audit results

**Fig. 1:**
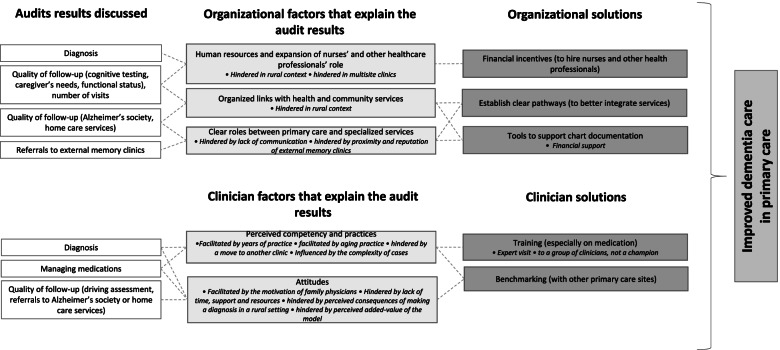
Summary of the discussions and links between results, insights, and solutions. This figure summarizes the audit results most discussed by the participants, their insights on organizational and clinician factors and their proposed solutions to improve dementia care in primary care. Modifiers that influence the factors and specifications as they relate to the proposed solutions are noted in italic. Dashed lines indicate participants’ explicit links between audit results, factors, and solutions

Three organizational factors were described by the participants to explain their audit results, namely a) human resources and expansion of nurses and other health professionals’ role, b) clear and organized links with health and community services, c) clear roles and support from external memory clinics. As depicted by the dash lines in Fig. 1, each organizational factor was linked (explicitly by the participants during the discussions) to two audit results, and alternatively, each audit result was linked to a maximum of two organizational factors. Furthermore, participants identified four modifiers that influence these organizational factors (rural settings, multisite clinics, poor communication, and proximity/reputation of external memory clinics were all hindering the organizational factors).

#### Human resources and expansion of nurses and other health professionals’ role

When discussing their audit results in general but mostly in terms of quality of follow-up (e.g., cognitive testing, caregiver’s needs, and functional status), number of visits, and making diagnosis, participants explained their results by the availability of human resources and the ability to use these resources to support the management of dementia. These resources, such as service coordinators and social workers, allowed clinicians to provide better care and support to persons with dementia by taking the brunt of the work. As such, the participants reported that having these resources enabled them to offer comprehensive assessment, treatment, and follow-up of persons with dementia, including the involvement of caregivers:

*[The clinicians from the collaborative memory clinic] do all the heavy lifting and give me advice and it certainly changes the way. Whereas, before I referred them to [external resources]. […] The quality of information I'm getting is much better than what we've had previously, more timely, and more cognitively-based.* - Site 107

In embedded-assessor sites, where a nurse or other healthcare professional performed cognitive assessments, the participants explained their audit results in general by the contribution these assessors made throughout the care process: from diagnosis to follow up. When discussing the diagnosis process, participants reported that the presence of the embedded assessors improved the timeliness of services and was perceived as an essential support for the adequate management of dementia:

*Generally, most people come through. So, they'll have the cognitive testing with [the nurse], while the caregiver sits with the social worker to do that caregiver interview […] So, they're timed. And then, we come together as a group to discuss the results.* – Site 107

Not all participants benefitted from human resources equally, especially in rural settings.

*Because of our [rural] location, I will say it to you: as a nurse practitioner in the office, I'm there three days a week, that's all the funding we have, our nurse is also there three days a week, so she's half-time, right? So, if we had her four days a week, you know, there would be more opportunity for her to take on some more projects.* – Site 103

Alternatively, participants, especially in multisite clinics (e.g., 105 and 106), explained that the presence of these human resources, especially dementia-specific resources such as the embedded-assessor or the collaborative memory clinic, also generated a high demand of referrals from family physicians, hindering the quality follow-up or their audit results in general. In consequence, they reported, the wait times for diagnosis or follow-up evaluation suffered. The participants added that this led some clinicians to make multiple competing referrals, to collaborative memory clinic, embedded-assessors, and to external memory clinics, to obtain timely access to services: *“Some people [refer] to both [collaborative memory clinic or external memory clinic], and they’re hoping whichever is first. It becomes an issue.”* – Site 106.

#### Clear and organized links with health and community services

Participants highlighted that having a good relationship with community services (such as the Alzheimer Society and home care services) helped them ensure that persons with dementia are indeed referred to these services. This close relationship, participants reported, also contributed to the timely and relevant sharing of information:

*The [Alzheimer Society] will fax [patient data] over to me and I will have to scan them into the EMR [electronic medical records]. They are using clinical judgement as to whether or not they feel that it is something that the physician should be made aware of. So, they won't always send it, but they do send it if they feel anything is pertinent and relevant. […] The social worker on our team is with the Alzheimer society.”* – Site 105

However, participants from rural communities reported a lack of access to these community services resources and long waiting lists due to higher proportion of persons living alone: *“[We have more people living alone in our region] because we’re so rural. There may be a lot of people who don’t have family around to help support […] Although we do have trouble with continuity and accountability.”* - Site 107.

#### Clear roles and support from external memory clinics

When discussing the audit results with regards to Alzheimer’s societies and home care services, participants also reported the importance of clearly defined roles for family physicians and memory clinic specialists. More specifically, they reported feeling that memory clinic specialists were better equipped than family physicians to support families and caregivers and deferred to specialists to establish a contact between patients and community services:

*I think in that kind of situation, there is some assumptions that [the external memory clinic] is also connecting them with the Alzheimer's society. Patients are going from the country to the city to go to [the external memory clinic], the Alzheimer's society is in the city, you know.* – Site 103

When discussing audit results as they relate specifically to referrals to external memory clinics, participants reported a lack of information and suboptimal communications:

*I really didn't get as good information as I'd hoped from the [external memory clinic]. […] So, when we started up our [collaborative memory clinic], the quality of the information is just that much superior to the information I'd been getting previously.* – Site 107

Alternatively, participants reported that the presence and proximity of well-established external memory clinics were driving higher referrals, prompted by the persons with dementia directly:

*Often the patient or provider ask about it. They know that memory clinics are available in this area. They love the quality of them. I think it's fairly well-known in the community. And they ask [for it], as soon as they hear we are going toward the dementia diagnosis, [they say]: ‘I need to go there and get the best treatment’, you know*. – Site 104

#### Clinicians’ factors that explain the primary care sites’ audit results

Participants offered three clinician-level factors that explain many of their audit results, namely a) perceived competency and practices, and b) attitudes. As depicted by the dash lines in Fig. 1, some audit results (namely diagnosis) were linked (explicitly by the participants during the discussions) to both factors, displaying good overlap between audit results and clinician factors. Furthermore, eight modifiers that influence these clinician factors (see Fig. 1) were mentioned by the participants: a) competency with diagnosis and medication management that evolves with years of practice or a move to another clinic and practices for diagnosis (and reliance on external memory clinics for guidance) was influenced by the complexity of cases, and b) attitudes toward diagnosis, medication managements and quality of follow-up relate to the clinicians’ motivation, lack of time, support and resources, and perceived consequences of a diagnosis as well as perceived added value of the model when it relates to referrals to external memory clinics.

#### Perceived competency and practices with regards to the care of persons with dementia in primary care

When discussing their audit results with regards to making a diagnosis, participants reported that this was supported by cumulated years of experience with older populations. This gradually built-on expertise made them feel confident with dementia care in general, including their perceived competency in making a diagnosis:

*Especially because we have a big senior's population and many of our physicians also work in long term care, or complex continuous care, or the rehab unit, the hospital. That is a really big part of their practice and they have probably developed competencies around those areas. And so they feel comfortable managing that piece, like making the diagnosis.* – Site 105

However, when discussing their audit results as it relates to managing dementia medications, participants expressed doubts and felt inadequately supported and uncomfortable doing it themselves and preferred referring patients to external memory clinics:

*Each drug, you think, they might have all kinds of side effects and so it was very difficult to make a decision. And to be honest, I very rarely know because of quality of back-up that we have locally. I just find it much easier to sit back long enough […] Sitting back, letting the experts carry on is a good idea.* – Site 104

When discussing making the diagnosis and managing dementia medications, the participants also reported that this could be influenced by an aging population in their clinical practice or changed due to patients moving from their practice to another one. Participants also reported how this could lower their confidence in making diagnosis and managing dementia medications:

*I was practicing outside of [the region], I didn't have the numbers of geriatric patients that I have out here. So, my practice now is very geriatric-heavy and so I am starting […] doing like the testing and stuff for us. So, I'm starting to start prescribing but I must say that, even me, I have been referring a fair amount to the [external memory clinic] because I just haven't prescribed any of those meds for years […].* – Site 104

When participants discussed their audits results as they relate to referrals to external memory clinics, they reported referring to obtain additional clinical guidance, where doubts persisted after their assessment or for more complex cases, reflecting good practice: *“That must have been like: ‘I think this person has dementia, do you agree?’. It wasn’t like: ‘what’s going on with this person?’ […] It was confirming the diagnosis.”* – Site 107.

#### Attitudes toward dementia care in primary care

Participants attributed their capacity to diagnose dementia and manage medications to the positive attitudes of their motivated family physicians: *“I think we have way more physicians that want to be involved in that process with their patient*s.” – Site 105.

But not all participants agreed on the positive attitudes of the family physicians working in their site. Some family physicians, the participants reported, were still reluctant to treating the dementia population due to lack of time, support, and resources: *“I think that family doctors generally want to take care of their patients, but with dementia it becomes very time consuming very quickly.”* – Site 101.

Despite these positive attitudes, participants reported many persisting negative attitudes, especially toward the management of dementia medications (whether patients had received a first prescription in primary care or from a specialist):

*I have hesitation with the medicine because the effect of this is not so great: side effects are great, and the evidence that it's going to stop the process is very small. So, if a person in my practice is put on medication, it's because the [external memory clinic] says “Yes, they qualify”. So, I kind of let them manage the meds although I always ask question because face it, we need better drugs for it.* – Site 103

In addition, participants reported negative attitudes toward making a diagnosis as in they perceived a diagnosis had more dramatic consequences due to their rural situation:

*And to be quite honest, because Alzheimer's and dementia is such a devastating diagnosis in the rural area: you have to move, you have to sell your car, you have to sell your house, your family has to come picking at your fortune, right? It's such a devastating diagnosis. I tend to not make that diagnosis myself. […] I have a big problem telling people they have Alzheimer's. […] Because it's a big game changer out where we live. There is no coming back. […].* – Site 103

Finally, when discussing their audit results on referrals to community services and external memory clinics, participants reported that some family physicians in their site kept referring to external memory services because they did not see the added value of the collaborative memory clinic and kept referring to well established and trusted external memory clinics: *“I feel like some of our physicians […] don’t feel the [collaborative memory clinic] people are specialists per se. They don’t feel that, perhaps, they are any more trained than they are.”* – Site 105.

#### Third sub-objective: Describe the solutions proposed by the participants to improve dementia care in primary care

When discussing their insights that can explain the audit results, participants offered five solutions to improve dementia care. As depicted by the dash lines in Fig. 1, each organizational factor was linked explicitly during the discussions to one or two organizational solutions. The solutions paralleled the organizational and clinician factors (both organizational and clinician solutions were offered) and two solutions (i.e., training and tools to support documentation) led to specifications on their delivery.

#### Financial incentives to support dementia care in primary care in general (organizational solution)

In response to the perceived lack of resources, some participants suggested additional funds for dementia care, in the form of financial incentives. This would in turn improve their audit results as they relate to quality of follow-up, diagnosis, and number of visits: *“I mean money’s always good. (laughs) Like in terms of, like, physician compensations, [it’s] not great for the [collaborative memory clinic], right?”* – Site 108.

These financial incentives, some participants reflected, would allow hiring more nurses or other health professionals to address dementia care specifically, or expand the role of existing resources, such as nurses dedicated to other chronic diseases.

*So, if a nurse does a cognitive test because her salary comes from the family health team, but she does cognitive tests and does care on one of my patients, I can’t bill any funding to that because the government already paid her salary to do that.* – Site 102

#### Clear pathway across dementia care services, including within their primary care site as well as with external memory clinics and community services (organizational solution)

With the variety of available services internal or external to their site, such as collaborative memory clinics, embedded-assessors, and/or external memory clinics, some sites witnessed confusion in the process of referring and following-up on the care of persons with dementia. To ensure integration of all services, participants suggested creating clear pathways:

*So, why couldn't each of the clinics, so the [collaborative memory clinic], the [external memory clinics], give a criterion to the physicians and nurse practitioners to say like an algorithm. So, if your patient presents with A, B and C, they go to the* [28] *memory clinic. If they present [with E, D, and F], they go to [external memory clinic].* – Site 106

This idea has been already implemented successfully in some sites where many services are already available and referred to it as a *“triage system”* (Site 105) in a referral form. These pathways, the participants suggested, would support links with community services and external memory clinics to improve quality of follow-up and referrals to memory clinics.

#### Improve chart documentation and use of prompts in patients’ charts (organizational solution)

To improve their link with both community services and external memory clinics, participants suggested that tools to support chart documentation, such as prompts, be used. This would in turn improve the quality of the follow-up and referrals to external memory clinics. Participants acknowledged the necessity of further financial resources: *“If it happens without me thinking about it. […] Right, if it was like an automatic tick box: ‘yes, we need to bring the Alzheimer’s society in somehow’. […] Someone makes sure Alzheimer’s society is called.”* – Site 103.

#### Training for dementia in primary care, especially for management of dementia medications (clinician solution)

Training, especially on medications, was one of the most expressed needs/solutions across sites. According to participants, not all family physicians feel competent to manage medications. The participants mentioned that training could take the form of an expert who visit the site and discuss their concerns:

*For me, it would probably be more around the medications, and you know, what to follow-up on and how often we have to. […] The medication monitoring - to get me more comfortable dealing with that. Like I don't think it's the diagnosis part.* – Site 104

In addition, the participants suggested that training could happen through the exchange of expertise among family physicians within their site, akin to the idea of a community of practice.

However, some participants expressed that training a champion might be time consuming and rather suggested that the responsibility and expertise be shared among all family physicians and nurse practitioners in their site:

*The other issue is if you have a physician champion, you know, how do you find someone who’s going to be engage in each group, who has the time to do that, because they also have their own practice usually and I would think that that would be, that could be a challenge in a lot of groups, there may be some groups where they’re fortunate and have a particular physician who has a keen interest in doing that but I think there’s going to be a lot of groups where you’re going to have trouble finding someone who’s going to take on that role.* – Site 102

*Or another way to do that is to raise the knowledge and the capacity, the competency of all physicians and nurses, and NPs.* – Site 101

#### Benchmarking with other primary care sites (clinician solution)

Participants expressed that the A&F process was insightful and that they would appreciate sharing with other sites their experiences with dementia care or their innovations to improve dementia care. They expressed wanting to learn about the successes and challenges of other clinics: “*Because we don’t know what other people are doing, so it’s interesting to know [what] other people are thinking.*” – Site 107.

## Discussion

The primary care clinicians, managers, and staff participating in the focus group discussed many audit results, shared their insights on multiple organizational and clinician factors that could explain their results and proposed solutions aligned to their insights. The insights and solutions were nuanced with contextual details to make the models of dementia care sustainable or to scale-up and disseminate these models to other interdisciplinary primary care sites.

Although many of the audit results discussed by the participants referred to physicians’ activities (i.e., diagnosis, referrals to external memory clinics, medications), possibly due to the fact that there were more physicians than other healthcare professionals taking part in the discussions, activities shared with other health care professionals (especially the quality of follow-up: with cognitive testing, functional status, caregiver’s needs, Alzheimer’s societies, and home care services) also solicited their fair share of discussion. This may reflect the general willingness of the participants to collaborate within an interdisciplinary practice [29].

During the discussion, the participants shared their insights on the factors that could explain their audit results, which in turn, facilitated the identification of possible solutions and areas of improvement in dementia care and their dementia care model. Such a reflective practice allowed the participants to take a logical approach to finding solutions [30].

The solutions generated by the participants echoed the same two levels as the factors they perceived as explaining their results. Both organizational- and clinician-level solutions were offered for the organizational and clinician factors, suggesting that their insights and solutions are well aligned to one another. Yet, many of the solutions proposed have already been explored in the literature.

For instance, clear pathways across dementia services to provide integrated care with a clear knowledge of who does what, when and where is indeed essential for quality of care [31]. Integration of care with external services is in line with the Canadian dementia guidelines, which posits primary care at the heart of dementia care, with specialists and tertiary care services as essential support when needed. Agreements and clear pathways facilitate specialists’ and community services’ support to primary care clinicians [32]. Similarly, financial incentives for dementia care have been associated with improved dementia care [33]. Training, especially in the form of interactive, repeated discussions between academic groups, professional associations and healthcare institutions, has been shown to improve care [34]. Furthermore, training could promote a positive shift in the attitudes of clinicians [35, 36]. A&F and benchmarking have been shown to have a positive impact on clinical practice [13]. Through increased awareness and motivation [13], benchmarking could lead to better detection, diagnosis, and management of dementia in primary care and improved overall dementia care. And finally, chart documentation tools, such as prompts, are essential to interdisciplinarity by increasing awareness [37], decision-making and communication [38], which in turn improves quality of care [31, 39]. Electronic chart reminders, indeed, may require higher resources for their implementation (e.g., funds, time, training), but may also lead to high return on investment [40]. Furthermore, they have been shown to improve adherence to guidelines, and thus improve quality of care, especially if an action-response is solicited from the clinicians [41–43]. Chart documentation also supports the integration of appropriate care pathways through complete, detailed, accurate and comprehensive referrals, to ultimately improve the quality of care [44].

However, despite this knowledge, family physicians are still perceiving low levels of competency for managing medications and despite past financial support, human resources are still lacking, and nurses and other health professionals’ roles is till limited to support the diagnosis and ensure the quality of follow-up. This could mean that new solutions or specifications to these solutions need to be explored. One of these specifications on a known solution was offered by the participants of our study, namely training a group of family physicians and nurse practitioners instead of limiting the training to a champion.

Some solutions were linked with multiple factors, which were in turn linked to multiple audit results. Therefore, targeting these solutions that affect multiple factors and multiple results may have more significant impact.

Finally, attitude, a factor explicitly linked to several audit results by the participants, was only loosely linked with one solution: benchmarking. No clear link appears to be established between benchmarking and attitudes improvement in the literature. Yet, it could be a vector of change through self-reflection benchmarking elicits [45], similarly to what has been previously demonstrated with reflective practice [46, 47]. Benchmarking often yield known solutions, as per our participants offered, but has been shown to renew healthcare professionals’ interest, foster a competitive spirit and promote active engagement [48]. Nonetheless, the loose link between attitudes and benchmarking by the participants of our study may explain persistent negative attitudes among some primary care clinicians.

### Strengths and limitations

We used a causal pathway models’ theory to describe the participants’ responses when presented with results and how these relate to the solutions they propose. Linking solutions to insights and results increases the likelihood that the solutions proposed by the participants achieve actual change and is most informative of how to improve the models of care implemented [26, 49]. Our study is a first attempt at linking results, insights, and solutions. As more studies are published, aligning solutions to insights may increase the success of these solutions to improve practices in dementia care [26]. Furthermore, our study supported a variety of models of dementia care in primary care, and fostered solutions potentially adaptable to more than one model [49]. Finally, we followed several strategies to ensure trustworthiness (credibility, transferability, dependability, and confirmability) of our study [27].

On the other end, our study has limitations that require attention. The choice of the sites and the focus group method had influence over what the participants reported. We purposely chose interdisciplinary primary care sites that had previously implemented an innovative primary dementia care model, were knowledgeable about existing dementia guidelines, and were motivated to improve dementia primary care. As such, our study may not be transferable to primary care sites that have not yet implemented a dementia care model for their practice.

The choice of focus group methods also influenced what the participants discussed. By grouping managers, physicians, other health professionals, along with administrative staff, may have prevented some to share their concerns openly. However, focus groups support a broad perspective exchange that is validating for the participants [50], and foster a common ground to facilitate organizational change [51], thus enriching our study. Other methods of data collection (e.g., ethnographic studies) may bring forth different and complementary information.

Similarly, the fact that there were more family physicians who took part in the discussions, may have skewed the discussions toward one group’s interests over the others. However, many of the discussed audit results concerned the activities of other health professionals, such as nurses, occupational therapists, and social workers, not just family physicians.

As we wanted to avoid power imbalance, we did not invite decision makers to the feedback and focus group discussions. This may explain why participants did not explain their audit results or offer solutions at the system level. Excluding external decision makers, however, allowed for more open discussions among the primary care members. Solutions for the improvement of dementia care models implemented occurring at multiple levels – at the clinician, the organization, and the system level – may have a wider and deeper reach [52]. Future studies could consider including external peers in the discussion groups. Future research may also consider using different data collection methods, such as ethnographic observational studies or including decision makers or external peers in the discussion, to offer more diverse perspectives, not limited to the participants’ reporting.

Another limitation is that we only used audio recordings, which limited our capacity to differentiate the participants within each focus group. However, given that our unit of analysis was the focus group and not the individuals participating, this limitation did not have an impact on our analysis.

## Conclusion

Overall, participants from the eight Ontario primary care sites that had implemented a dementia care model offered a rich array of solutions to improve dementia care in their practice. These solutions were explicitly linked to the participants’ insights into the factors that explain their audit results. The deep and nuanced solutions offered in our study could guide decision makers to better support sustainable primary care sites in improving dementia care. This is especially important for government-level decision makers when considering the implementation of an Alzheimer Plan, such as the Ontario Alzheimer Plan or the Canadian Dementia Strategy.

## Supplementary Information

**Additional file 1.** Timeline of study and description of dementia care models.

**Additional file 2.** Template of feedback presentations.

**Additional file 3.** Focus Group discussion guides developed for this study.

## Data Availability

The datasets used and/or analysed during the current study are available from the corresponding author or the last authors on reasonable request.

## Abbreviations

A&F: Audit and feedback
CIHR: Canadian Institutes of Healthcare Research
CCNA: Canadian Consortium on Neurodegeneration in Aging
KAP: Knowledge, attitudes, and practices
BPSD: Behavioral and Psychological Symptoms in Dementia
EMR: Electronic medical records
