# Supplementary material for: Improving dementia care: insights from audit and feedback in interdisciplinary primary care sites
Source: BMC Health Serv Res. 2022 Mar 17;22:353. doi: 10.1186/s12913-022-07672-5 (PMC8931981; doi:10.1186/s12913-022-07672-5)
Supplement: Supplementary file 1 — Additional file 1. Timeline of study and description of dementia care models. [file 12913_2022_7672_MOESM1_ESM.docx]

Additional file 1. Timeline of study and description of dementia care models


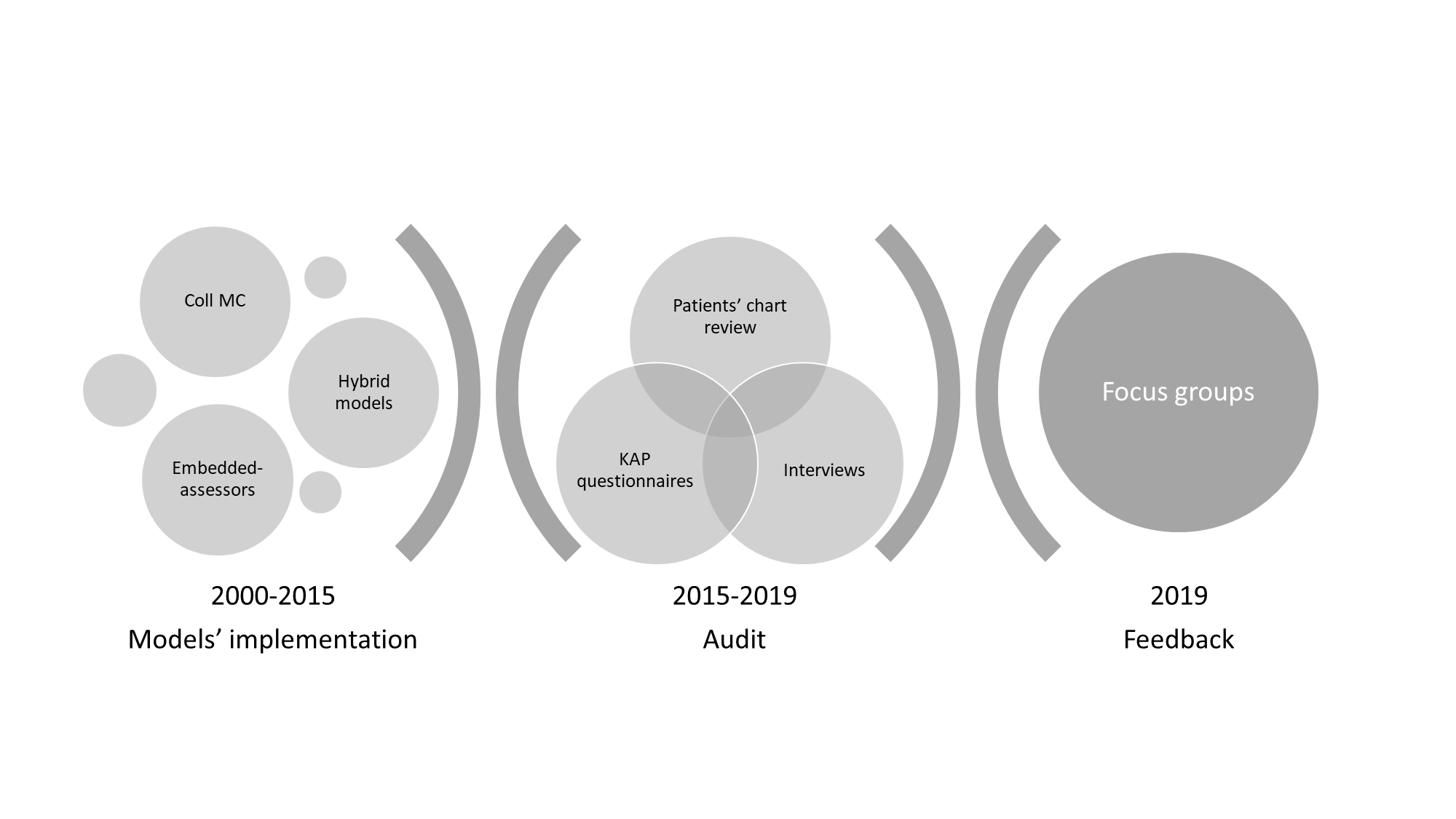
The current study stems from a large, 3-province, CIHR-funded study (described in Vedel et al. 2020, BMJ Open). This graph depicts the timeline of implementation, audit, and feedback sessions for the 8 primary care sites included in the current study.

« **Models’ implementation**»

Three different dementia care models were implemented in the selected 8 interdisciplinary primary care sites in Ontario between the early 2000’s and 2015: namely, collaborative memory clinics, embedded-assessors, and a hybrid model of both. In 2015, our team approached the primary care sites where a primary care model of dementia care had been developed to participate in our larger, 3-province study.

- A collaborative memory clinic model within primary care (Coll CM) which consists of a specially trained multidisciplinary team, including family physicians, nurses, social workers, pharmacists and occupational therapists (as available) as well as the presence of community agencies, all working together concurrently (34).
- An embedded-assessor model where a trained nurse works in partnership with family physicians to detect, evaluate, and manage patients with dementia and their family, while connecting them with community services, such as the Alzheimer’s Society. The embedded-assessor model can also include other healthcare professionals, mainly a social worker, and could also include occupational therapists, pharmacist, and psychologist.
- A hybrid model where both the collaborative memory clinic and the embedded-assessor model co-exist.

In these three models, the primary care sites were supported by external memory clinics, such as the Geriatric Assessment and Intervention Network Clinic (GAIN) sites.

« **Audit** »

The audit consisted in a convergent mixed-methods study design and occurred between 2015 and 2019, as per the larger study protocol.

1) An observational study with a cross-sectional design using a retrospective patients’ chart review and a validated questionnaire for family physicians was conducted.

a) In each site, the medical charts of 35 patients (75+ years old) per site with a diagnosis of dementia and who had at least one visit to the site during a 9-month review period (1 October 2015 to 1 July 2016) were reviewed. The primary outcome was a validated score on the quality of follow-up comprised of 10 indicators of adherence to Canadian guidelines in terms of performing and documenting in the patients’ chart specific evaluations (i.e., cognitive status, functional status, behavioral and psychological symptoms of dementia, weight, caregiver needs, driving status, home care needs, community service needs, dementia medications, and the absence of anticholinergic medication). Secondary outcomes that relate to continuity of primary care (e.g., number of visits to the site; the number of notes recorded in the charts); and to medications management (e.g., proportion of patients with dementia treated with dementia medications; proportion of new dementia medications initiated by family physician at that site; proportion of new dementia medications initiated by specialists; and proportion of patients treated with antipsychotics during the study period) were also assessed. Patients’ characteristics (age, number of women, and living status of patients) were also measured.

b) Furthermore, one validated Knowledge, attitudes, and practice (KAP) questionnaire was distributed to all family physicians working at the 8 sites between 2016 and 2018. This questionnaire assessed the physicians’ perceived competency and knowledge related to dementia; attitudes towards dementia; practices in terms of cognitive evaluation; attitude towards their collaboration with other healthcare professionals at the site; and attitudes towards the model of dementia care implemented.

2) A qualitative descriptive study using semi-structured interviews was concurrently conducted to examine the implementation of the dementia care models.

Semi-structured interviews were conducted between 2017 and 2019 with a convenient sample of three clinicians (one family physician, one nurse and one other health professional) involved in delivering care and with the geriatrician or family physician leaders (JI and LL both co-authors on the current study) who implemented the dementia care models within each site.

« **Feedback**»

The feedback was conducted during the summer of 2019. As per the larger study, an integrated knowledge exchange strategy was used to ensure uptake by principal stakeholders throughout the research, however the feedback sessions of the current study were an addition to the larger study. Our research team, thus conducted eight focus groups, presenting personalized audit results to each site in comparison to the other sites. All staff, clinicians and managers from the sites were invited to the feedback sessions and to participate in the discussion, which constitutes the data for the current study.
