# Supplementary material for: Improving dementia care: insights from audit and feedback in interdisciplinary primary care sites
Source: BMC Health Serv Res. 2022 Mar 17;22:353. doi: 10.1186/s12913-022-07672-5 (PMC8931981; doi:10.1186/s12913-022-07672-5)
Supplement: Supplementary file 2 — Additional file 2. Template of feedback presentations. [file 12913_2022_7672_MOESM2_ESM.docx]

Additional file 2. Template of feedback presentations
