# Supplementary material for: Improving dementia care: insights from audit and feedback in interdisciplinary primary care sites
Source: BMC Health Serv Res. 2022 Mar 17;22:353. doi: 10.1186/s12913-022-07672-5 (PMC8931981; doi:10.1186/s12913-022-07672-5)
Supplement: Supplementary file 3 — Additional file 3. Focus Group discussion guides developed for this study. [file 12913_2022_7672_MOESM3_ESM.docx]

Additional file 3. Focus Group discussion guides developed for this study

Following a short presentation of their results, discuss the following questions as a group:

1. Which results did you find useful / interesting / expected / surprising?
2. What are the key elements that explain these results?
3. What changes in terms of results would you like to see when this evaluation is performed in the future?
4. What would help your site to achieve these changes/goals?
